# Spatially-resolved optical monitoring of bioreactor cell growth

**DOI:** 10.1364/BOE.583724

**Published:** 2026-01-30

**Authors:** J. Gorecki, C. Redwood-Sawyerr, J. Cao, H. Dehghani, C. Kontoravdi, K. Polizzi, C. Rowlands

**Affiliations:** 1 Department of Bioengineering, Royal School of Mines, Imperial College London, SW7 2BX, UK; 2 Department of Chemical Engineering, Royal School of Mines, Imperial College London, SW7 2BX, UK; 3 School of Computer Science, University of Birmingham, B15 2TT, UK

## Abstract

Bioreactors are used for the industrial-scale culture of cells to obtain valuable products such as pharmaceuticals, enzymes, and biofuels; however, monitoring the growth conditions within the vessels is challenging and is often dependent on single-point ex-situ measurements. Further, spatial heterogeneities are known to exist within these environments, thereby creating regions of low growth or incomplete reactions, reducing yield, and importantly, reducing the applicability of single-point measurement methods. Optical imaging is an attractive method for remote spatially-resolved measurement platforms; however, the strong optical scattering within cell cultures makes imaging almost impossible. Here, we utilise this parasitic scattering effect and present a spatially resolved optical method for monitoring cell density within a bioreactor, using optical measurement of local scattering parameters as a proxy measurement for cell density. Our method is non-invasive and does not require the removal of any cell material from within the vessel. We propose that our optical measurement method can be incorporated into process-control feedback systems, providing insightful information on cell growth that can be used to deliver higher spatial homogeneity and increased yields.

## 1. Introduction

Bioreactors are vessels designed to sustain a large-scale cell culture, the purpose of which is to produce commercial-scale quantities of a biological product. Bioreactors encompass a wide range of size scales and applications, such as; pharmaceutical synthesis [1], tissue culture [2], the production of food and beverages [3], and sewage treatment [4]. In all cases, the bioreactors rely on biological agents such as bacteria, fungi, or mammalian cells, to perform biological reactions with yields, reaction conditions or cost that cannot be achieved by chemical reactors. Bioreactors play a critical role in modern industrial processing, such as in the pharmaceuticals industry; crucially, this industry requires stringent control over processes and outputs, as even small deviations in the composition of the product can lead to medicines that are either ineffective or cause adverse effects in patients. Further, due to the high costs involved in pharmaceutical products, any decreases in yield or wasted batches can prove extremely costly. However, contrary to these requirements, the interior conditions of bioreactors are complex and dynamic environments, involving mechanical movement; a mixture of gas, liquid, and solid phases; chemical gradients; and living organisms; each of which evolve over time in an interlinked and heterogeneous system [5,6].

Cell behaviour within bioreactors is known to be temperamental and unpredictable, and to make matters worse, there are practical limitations that make monitoring the cells and environmental conditions a challenging task. In-depth review articles have been published discussing the heterogeneities present in bioreactors [5,6]. We highlight some of the key information from these reviews to provide a general overview of the types of heterogeneities which may appear in bioreactors. **Mechanical Agitation** of the bioreactor contents driven by rotation of an impeller is the main method of mixing the vessel contents. Agitation aims to homogenise the environmental conditions of the bioreactor, however the maximum rates of agitation are limited due to the shear-stress applied to the cells. Further, the stirring rates within bioreactors are known to be dependent on spatial location; a study utilising particle imaging velocimetry found that fluid compartmentalisation effects can be produced, where regions of the vessel experience low mixing rates with other regions, due to the agitation dynamics [7]. Incomplete mixing will therefore lead to heterogeneities created within the vessel which will influence cell growth. **Dissolved Oxygen** concentration gradients within bioreactors often appear as oxygen exhibits a low solubility in water, and therefore when the oxygen supply is locally exhausted by aerobic processes it can be slow to equilibrate [8]. **pH Gradients** are easily formed due to the production of acidic by-products. Further, many cell functions are influenced by pH, making it important to monitor and maintain the homogeneity of pH within the reactor. Spatial distribution of pH has been measured by a variety of methods, such as introducing phenolphthalein into the vessel, or mechanically moving an electrochemical pH probe [9]. **Carbon Dioxide** is released from cells during respiration, and dissolves into the surrounding medium due to the high solubility of carbon dioxide in water. Dissolved carbon dioxide (dCO_2_) is removed from the bioreactor vessel by the sparger, which releases gas bubbles into the vessel, allowing the dCO_2_ to diffuse from the liquid into the gas bubble and be carried upwards to the surface. The solubility of carbon dioxide in water is altered by pressure, which depends on the height of liquid in the vessel. For tall vessels this can create a gradient along the vertical axis [10]. **Shear Stress** is experienced by cells due to the movement of liquids in the surrounding media, with the main contributions arising from the movement of the impeller [11] and gas bubbles [12]. **Nutrient** gradients can arise where the local consumption exceeds supply. Nutrients contained in the cell media generally have a high degree of solubility in water, especially compared to oxygen, and therefore are not assumed to be a rate-limiting parameter [13], however this assumption will depend strongly on the agitation and mixing dynamics within the system. The presence of parameter gradients in bioreactors can lead to variances in yield [14], gene expression [15], and mutation rate [16]. Further, the presence of heterogeneities in bioreactors creates additional challenges from a process monitoring and modelling perspective, as models must be adapted to include additional parameters, thereby increasing modelling complexity [17].

To overcome these issues, adequate process monitoring is essential, in order to investigate, control, and alleviate the inherent heterogeneity inside bioreactors. Bioreactor process monitoring can be achieved through a wide range of methods, and a wide range of parameters are amenable to measurement [18,19]. These processes can be categorised into *in-situ* processes, where contents of the bioreactor are monitored inside the bioreactor vessel, or *ex-situ* processes, where the contents are monitored outside of the vessel. Each category has its own advantages and drawbacks depending on the specific application. ***In-Situ* monitoring** allows the contents of the bioreactor to be observed in their native state, however gaining access to the materials can be complicated by the need to avoid contamination or changes inside the bioreactor. ***Ex-situ* monitoring** is generally easier to perform, as the sample can be easily manipulated, however by removing the sample from its native environment it may undergo changes after removal. The ideal process monitoring method is therefore broadly applicable to a wide range of processes, possesses sufficient spatial resolution to resolve the reactor heterogeneities, and is non-invasive, cheap and easy to use.

Here we present a method for investigating spatially-resolved optically-measured cell growth within a bioreactor. Our method is non-invasive and fully automated, thereby allowing for long-duration measurements to be acquired without affecting the bioreactor environment. The technique utilises the optical scattering which is induced in the liquid suspension due to the cells. By scanning the vessel with a laser, and imaging the spatial variations in scattered light within the vessel, we are able to extract local measurements of the effective scattering parameter, which is shown to be linearly proportional to cell density, within certain cell densities. By scanning the laser beam over the surface of the vessel we build up maps of local cell-density, thereby monitoring spatial heterogeneities within the bioreactor. The optical measurements are fast to acquire, and do not require any cellular material to be removed from the vessel. We demonstrate the measurement system by cultivating *E. coli* bacteria in the bioreactor vessel, and monitoring cell growth over a 23 hour period. Spectrophotometer measurements of OD_600_ (the optical density measured at 600 nm) from sample aliquots are provided as an independent measure of cell growth to verify our measurement method. Our ultimate goal is to combine this system with genetically engineered reporter genes which are able to provide spectrally resolved information on cell function. We envisage that this system can be used to easily monitor cell growth, and could be built into a process-control feedback loop to improve bioreactor homogeneity, thereby increasing reaction yields at very modest cost.

## 2. Methods

### 2.1. Cell preparation

#### Preparation of *E. coli* Cells for the Characterization of Culture Scattering Properties Within a Bioreactor

The pRSET-T3 plasmid (derived from the commercially available pRSET-A and encoding maltose-binding protein) was transformed into chemically-competent *E. coli* DH5*α* cells. Cultures were grown in Lysogeny Broth (LB) supplemented with the appropriate antibiotic ("complete LB media"). Unless otherwise specified, all incubations were performed at 37 ^∘^C with shaking at 250 rpm. An overnight starter culture (4 mL) was grown for 16 h in complete LB medium and used to inoculate 400 mL of complete LB medium in a 2 L baffled flask (1:100 ratio). After ∼16 h, the culture was centrifuged (3,220 g, 10 min, 4 ^∘^C), and the supernatant was discarded. The resulting pellet was resuspended to inoculate 2 L of M9 medium for spectrophotometric analysis in a bioreactor. M9 medium was supplemented with 0.3 mM CaCl_2_, 1 mM MgSO_4_, 1 mg/L thiamine, the appropriate antibiotic, and 0.4 % (w/v) D-glucose ("complete M9 media"). For a separate experiment, an 8 mL overnight starter culture was prepared under the same conditions and used to inoculate two 400 mL complete LB cultures in separate 2 L baffled flasks. After 16 h, both cultures were harvested by centrifugation (3,220 × g, 20 min, 4 ^∘^C). The combined pellets were divided into 16 fractions, which were used to inoculate 2 L of complete M9 medium for stepwise spectrophotometric analysis in the bioreactor.

#### Culturing Whole-Cell Lactate Biosensor Cultures for Fluorescence-Based Diffuse Optical Tomography Analysis

The plasmids pJ23118-LldR-GFP and pJ23118-LldR-GFP-J23100-mCherry-ldhL, which confer whole-cell L-lactate biosensor functionality, were transformed into chemically competent *E. coli* DH5*α* cells. A 4 mL overnight starter culture of each strain was grown for 16 h in complete LB medium, then transferred into complete M9 media and grown to an OD_600_ of 0.4 before use in functional bioassays. For bioreactor experiments, cells harbouring pJ23118-LldR-GFP-J23100-mCherry-ldhL were prepared following the same procedure described for pRSET-T3 cultures.

#### Antibiotic Selection and Growth Arrest Conditions

Cells harbouring pRSET-T3 were cultured in the presence of 100 *μ*g/mL ampicillin. Cultures containing pJ23118-LldR-GFP or pJ23118-LldR-GFP-J23100-mCherry-ldhL were grown in media containing 50 *μ*g/mL kanamycin. Where inhibition of cell growth was required without reducing cell viability, 25 *μ*g/mL chloramphenicol was used.

### 2.2. Optical setup

**Fig. 1.**
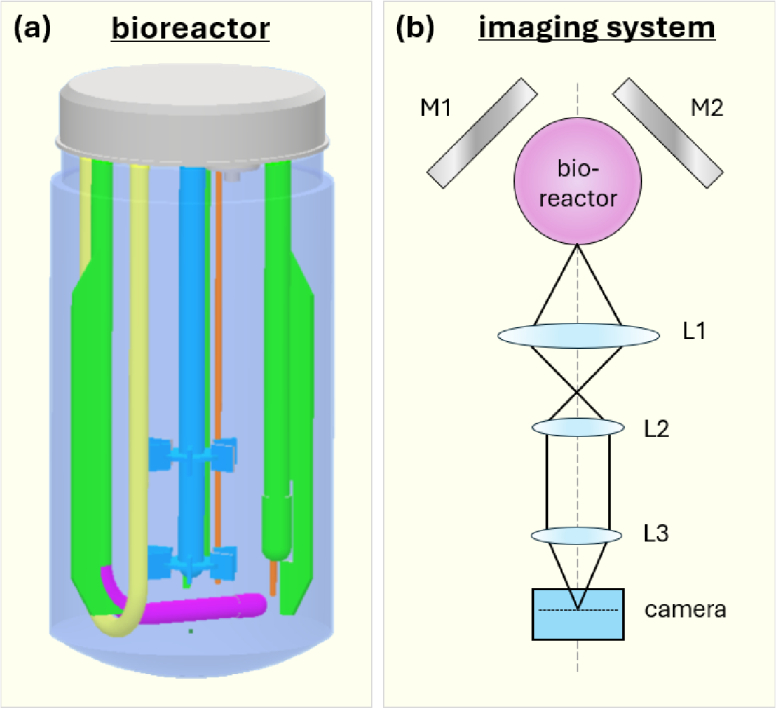
**(a) 3D model of the disposable bioreactor**, depicting the baffles (green), an agitator (blue), sparger (purple), heating loop (yellow), and thermometer probes (orange). **(b) Schematic diagram of imaging setup**; the bioreactor vessel surrounded by two mirrors to allow imaging of the front and rear surfaces. A large fixed-focal-length lens (L1) focuses the light which is collimated by lens L2 and then imaged into the camera sensor via lens L3. The imaging system is designed to create a collimated beam between L2 and L3 within which optical elements such as filters could be placed without introducing spherical aberration.

The experiment utilised a disposable bioreactor purchased from Applikon (Applikon Appliflex ST [20]) which is shown in Fig. 1(a). The bioreactor consisted of a 2.5 L cylindrical vessel, and a uni-body plastic manifold including baffles to create turbulence in the fluid, an impeller for mechanical agitation, heating loops to control temperature, and a sparger to provide gas exchange. An optical system was designed to allow for laser projection and imaging from all sides of the cylindrical bioreactor vessel.

An aerial view of the imaging system is provided in Fig. 1(b) showing the orientation of the bioreactor vessel and two flanking mirrors, allowing for illumination and imaging of the front and rear surfaces of the vessel. The imaging assembly consisted of a large fixed-focal-length lens (Edmund Optics #86-5690), labelled L1, with an 8 mm focal length and 100 mm to infinity working distance, allowing for imaging over a wide field of view. The second lens (L2) was a laser scanning tube lens (Thorlabs TTL 200-MP) with 200 mm effective focal length. Lens L3 was a 200 mm focal length tube lenses (Thorlabs TTL 200-A), aligned to form an image on the camera sensors. The distance between lenses L1 and L2, and lenses L2 and L3, was the sum of their focal lengths, thereby forming an image relay system. The image relay system produced a region within which optical elements such as filters could be placed (between lens L2 and L3) without encountering spherical aberration effects. All opto-mechanics of the imaging assembly were held within a Thorlabs 30 mm cage system. The camera was a monochrome sensors obtained from IDS Imaging (U3-3290SE-M-GL), containing 4096 x 2160 pixels covering a sensor area of 14.2 x 7.5 mm. The camera sensor noise was characterised via a temporal variance method [21] and detailed in Supplement 1 alongside analysis of the camera black level. The entire optical system is housed within a light-proof black box to remove the possibility of external light entering the system, or stray internal light from reflecting from the box walls. Further, the entire optical path between the imaging lens (L1) and the camera sensor is enclosed with aluminium lens tubes, and any empty faces on beam-splitter cubes were enclosed with aluminium blanking caps, to ensure stray-light causes negligible influence on the experiment. Lens L1 had a numerical aperture of 0.0174 which was the factor limiting the resolution of the imaging system. Based on an illumination wavelength of 473 nm the imaging system has a resolution, determined by the Rayleigh criterion, of 33 *μm*.

**Fig. 2.**
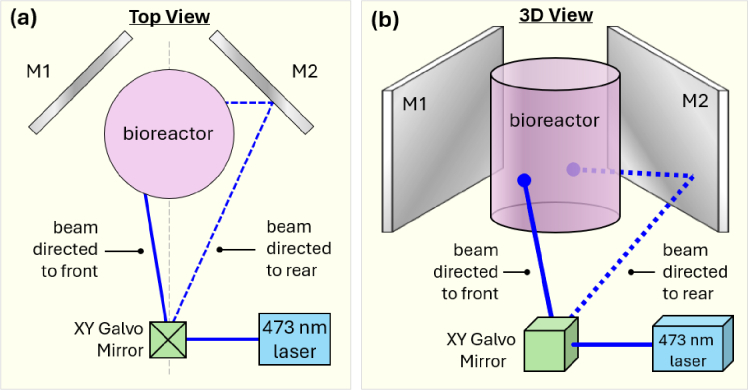
The laser projection system is shown in **(a) Top View** and **(b) 3D View**; the laser beam is able to be directed to any surface of the bioreactor vessel by the use of an XY galvo-mirror and two large mirrors flanking each side of the bioreactor. The laser optics are placed directly below the imaging assembly.

The laser projection assembly was held directly below the imaging assembly, and is shown separately in Fig. 2(a). The assembly consisted of a 100 mW continuous-wave blue laser at 473 nm (Omicron LuxX 473) which was directed into a 2-axis scanning galvo-mirror pair, allowing the laser beam to be steered in two axes. Two large flat mirrors (M1 and M2) were placed either side of the bioreactor vessel. The mirrors were oriented such that the laser beam could be steered towards any spot on the cylindrical surface, in both its circumference and height. The diagram depicts two examples of beam paths, showing how the laser can be incident directly on the front surface of the vessel, or to illuminate the rear surface via one of the mirrors. Figure 2(b) demonstrates an identical configuration in a 3D representation to aid visualisation.

### 2.3. Image transformation

**Fig. 3.**
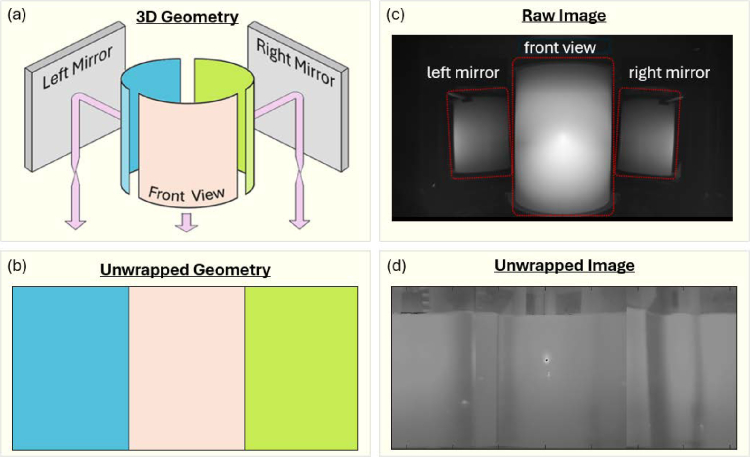
**(a)** The bioreactor vessel is imaged from three viewpoints; either directly on the front view, or on the two rear sections via the left and right mirrors. **(b)** A geometric projection algorithm isolates each of the three sections in the image and projects their curved view on to a flat surface, thereby reproducing the ’unwrapped’ image of the vessel surface. **(c)** An example raw camera image highlighting the front of the vessel alongside the two mirror images of the rear sections. **(d)** The raw image is transformed via the geometric projection algoritm to produce the unwrapped image.

The optical imaging system captures an image of the front of the cylindrical bioreactor vessel along with two images of the rear via the aforementioned two large mirrors placed on either side of the bioreactor. An algorithm was designed to isolate these three sections within the raw image and transform them into a flat projection of the cylindrical surface. As a simply analogy; if the cylindrical surface of the vessel were wrapped with a sheet of paper, the algorithm takes the three images of this curved sheet of paper and recreates how it would look if removed from the vessel and laid out flat. A schematic representation of this process is depicted in Fig. 3(a&b).

The main steps of the algorithm are as follows; 1) Isolate each of the three regions in the raw image via a predefined polygon. 2) Each of the three regions has a unique geometric transformation, utilising the Matlab function geometricTransform2d, which projects the curved image onto a flat surface. 3) The projections of each rear-vessel section are reversed in the horizontal axis due to being imaged via a mirror. 4) The three projections are stitched together to produce the final ’unwrapped’ image.

The imaging setup relies of acquiring sensor data over a wide range of intensity values and therefore to avoid issues of sensor saturation a high dynamic range (HDR) imaging protocol is utilised which is detailed in Supplement 1.

Figure 3(c) shows an example of the raw image from the blue channel camera during laser illumination of the bioreactor filled with a scattering medium. The three distinct regions-of-interest are outlined with red polygons as a visual aid. Each of these three regions must be isolated within the algorithm and processed individually as they each possess a unique geometric projection. Figure 3(d) depicts an example of an unwrapped image of the bioreactor. In the centre of the vessel the bright spot is due to the location of the laser. The dark band in the top section of the image is caused by the height of the liquid within the vessel, as the liquid is a scattering medium while the air above it does not induce any scattering. Several vertical dark bands are visible in the image which are due to the presence of the plastic baffles within the bioreactor manifold. Several of these baffles are visible in the image as they are located in close proximity to the edge of the vessel, and therefore the optical diffusion within the scattering media is not sufficient to obscure them.

### 2.4. Extraction of optical scattering parameters

Light passing through the vessel experiences both optical scattering and absorption effects due to turbidity in the culture medium. The contents of the vessel include water, cell media, and bacteria cells. The water and cell media both exhibit negligible absorption nor scattering, nevertheless the presence of the cells results in both absorption and scattering of light passing through the vessel. Decoupling the effects of absorption and scattering during the propagation of light is a non-trivial process, and therefore here we calculate a lumped parameter known as the ‘effective scattering parameter’. The effective scattering parameter $\mu_{\text{eff}}$ is a product of the absorption coefficient $\mu_{a}$ and the reduced scattering coefficient $\mu_{s}^{\prime }$ , shown in Eq. (1).

(1) $$\mu_{\text{eff}}=\sqrt{3\;\mu_{a}\;\mu_{s}^{\prime }}$$

For low cell concentrations we hypothesise that both the absorption and the reduced scattering coefficients are linearly proportional to the number of cells, and therefore the effective scattering coefficient should be linearly proportional to cell number.

The effective scattering coefficient can be extracted from the imaging data by analysing the image intensity **I** along a straight line, as a function of distance **d** from the laser illumination spot [22]. Equation (2) provides the relation between the image intensity, distance from the laser spot, and the effective scattering coefficient. The equation is derived for a semi-infinite space which is a suitable approximation when the bioreactor vessel is large and the media contained within is opaque. By plotting the natural logarithm of intensity multiplied by distance squared, against distance, a linear fit can be used to extract the gradient which is equal to the effective scattering coefficient; the intercept provides the extra term **k** which is unused.

(2) $$log(I\;d^{2})=-d\;\mu_{\text{eff}}+k$$

Due to the cylindrical shape of the bioreactor vessel, and the stirring of the liquid, we assume that the cell densities in the vessel will show negligible variation along the horizontal axis, but that there may be variations along the vertical axis. Therefore, we illuminate the vessel on the front surface, utilising 25 laser locations along a vertical line. The laser locations are equally spaced at 5 mm intervals, providing a coverage over 125 mm in the vertical axis. To analyse the effective scattering coefficient we extract the image intensity along a horizontal line, within a region of 5 to 30 mm distance from the laser location. The set of 25 images requires around two minutes to acquire, and is set in a loop to repeat at 10 minute intervals, generating 138 datasets over the 23 hour monitoring period.

## 3. Results

### 3.1. Relationship between cell density and effective scattering parameter

To verify that cell density within the cell culture medium produces a measurable change in the effective scattering parameter, a calibration experiment is performed. A large batch of E. coli cells was grown in 800 mL of diluted cell medium. Spectrophotometer measurements of OD_600_ are used to estimate the initial density of cells. An OD_600_ value for the cell suspension of 9.1 was measured, which equates to a cell density of 7.28 x10^9^ cells per mL of liquid [23], and therefore a total of 5.82 x10^12^ cells. The cells were centrifuged to remove the liquid, and were divided into 16 equal aliquots. Each aliquot is estimated to contain 3.64 x10^11^ cells. The bioreactor vessel is filled with 1.6 litres of water and 0.4 litres of cell media, and the impeller set at a constant stirring speed of 500 rpm. The cell aliquots were sequentially added to the bioreactor and the imaging routine undertaken for each. The addition of each aliquot will impose a negligible change on the total volume of liquid (2 litres total) and therefore the increase in the cell density within the bioreactor due to the addition of each aliquot can be calculated as 1.82 x10^8^ cells per mL. The gradient of the effective scattering parameter against the cell density determines the conversion from scattering parameter to cell density. Subsequently, the cell density values are normalised against the starting value at T = 0 to determine cell growth factors.

**Fig. 4.**
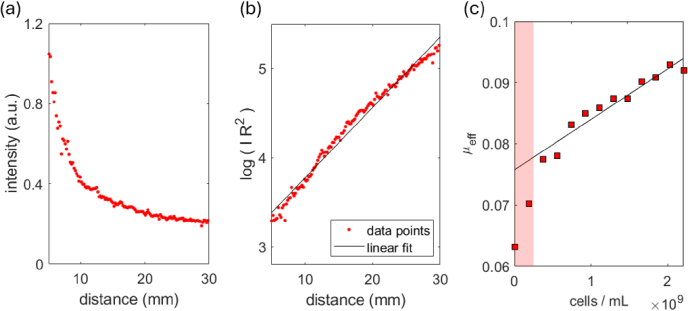
**(a)** The image intensity is analysed along the horizontal direction, and plotted as a function of distance from the laser spot. **(b)** The natural logarithm of the intensity is multiplied by the square of the distance, and then plotted against distance. The gradient of this linearised data provides the effective scattering parameters $\mu_{\text{eff}}$ . **(c)** Effective scattering parameter is obtained for a range of cell densities, and a linear fit applied to the data. The two data points in the highlighted region are excluded from the linear fit due to their low concentration.

Figure 4(a) plots an example of the image intensity as a function of distance from the laser spot for a given concentration of cells, analysed along a straight horizontal line. The intensity at the laser spot (d = 0) is cropped from the graph as it exhibits saturation of the camera sensor, and does not provide useful measurements. The data reveals the intensity decays quickly when near the illumination spot, however then tails off slowly at longer distances. In Fig. 4(b) the natural logarithm of the intensity multiplied by the square of the distance is plotted against the distance. As expected this product displays a linear relationship which is fit using a least squares method to extract the gradient and intercept. In this scenario the gradient of the linear fit provides that value of the effective scattering coefficient. To analyse how the cell number affects the optical properties, the effective scattering coefficient is plotted against the cell density in Fig. 4(c). To ensure that the calibration experiment covers an applicable range of cell densities that would be encountered in the bioreactor, the maximum density utilised in the calibration experiment is equal to 3-times the initial cell density that is used for cell fermentation experiments. The data reveals a linear relationship where the effective scattering parameter increases as cell number increases. Finally, a linear function is fit to the data in Fig. 4(c) to obtain a conversion between the measured value of $\mu_{\text{eff}}$ and the cell density ***ρ*** per mL of liquid, which is provided in Eq. (3). An R-squared value of 0.9322 is obtained for the linear fit. When performing the linear fit the two lowest concentration data points are excluded, which are highlighted in the red region. These low concentration data points are excluded because our imaging method relies on the presence of optical scattering, and therefore with non-scattering media the analysis method is not applicable. It was expected that this graph would show a linear trend with a near-zero value of $\mu_{\text{eff}}$ when the cell density is zero, as the water and cell medium should not induce scattering. In reality we find that even when the cell density is zero there is a non-zero contribution to the effective scattering parameter. This effect is likely due to scattering from the internal plastic manifold of the bioreactor vessel, and any surface imperfections on the outside of the plastic vessel. Regardless, both of these effects will remain fixed during the experiment and therefore are not a cause for concern.

(3) $$\rho [cells/mL]=\frac{\mu_{\text{eff}}-7.58\times 10^{-2}}{8.23\times 10^{-12}}$$

### 3.2. Spatial monitoring of cell growth

A natural strain of *E. coli* (without any genetic modification) is grown within the Applikon bioreactor in diluted cell media (consisting of 1.6 litres water and 0.4 litres of cell media). The bioreactor is at ambient room temperature, with the impeller stirring at a constant speed of 500 rpm. The gas sparger and various control loops are not utilised. The growth of the cells is monitored over a 23 hour period; spatially resolved cell growth information is obtained by scanning the laser along the vertical axis of the vessel and recording the scattering information at 25 discrete locations to extract the local value of the effective scattering parameter $\mu_{\text{eff}}$ . An independent measurement of cell density is provided via spectrophotometer measurements on 1 mL aliquots of OD_600_ which are taken at one-hour intervals for the first twelve hours, and then a final reading at end of the experiment. A negative control experiment is devised, employing *E. coli* cells cultured with an antibiotic to suppress cell growth, while spatially monitoring the effective scattering parameter within the vessel. It is expected that during the control experiment there will be negligible growth of the cells, and negligible change in their spatial distribution within the vessel.

Each aliquot is injected into a plastic cuvette and inserted into the spectrophotometer to measure the OD_600_, which quantifies the amount of light at 600 nm that is transmitted through the cuvette. Any light which is not transmitted must be either absorbed or scattered by the sample. At low cell concentrations the OD_600_ value is known to be linearly proportional to the cell density, and therefore is the gold-standard method for monitoring cell growth due it its simplicity and ease-of-use [24]. Nevertheless, this method has drawbacks, as it requires manually taking an aliquot from the vessel and does not convey any information on spatial heterogeneity, which is especially important for larger vessels.

**Fig. 5.**
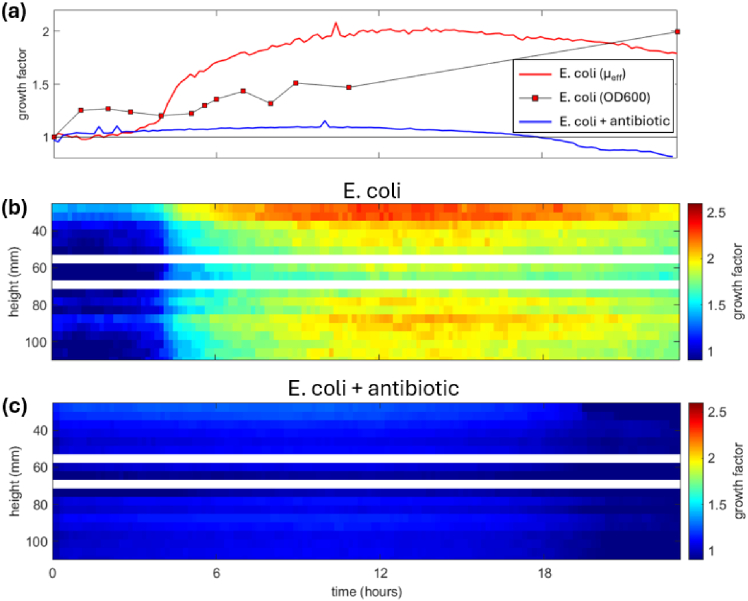
**(a)** Average cell growth within the bioreactor vessel for E. coli plotted in red, and E. coli + antibiotic (negative control experiment) plotted in blue. **(b)** Spatially resolved measurements of cell growth for E. coli reveal large variations in cell growth over the height axis of the vessel, suggesting regions of heterogeneity. **(c)** The negative control experiment (E. coli with antibiotic) reveals negligible spatial variations in cell growth, suggesting the vessel remains largely homogeneous in the absence of cell growth.

Figure 5(a) plots the average cell growth in the vessel for the E. coli cells (red line) and the negative control of E. coli cells with antibiotic (blue line), both obtained from the $\mu_{\text{eff}}$ scattering parameter measurements. The independent measure of cell growth from OD_600_ measurements for the E. coli experiment are overlaid in red square markers. All measurements are normalised against the readings taken at T = 0. The growth factor for the E. coli obtained from $\mu_{\text{eff}}$ measurements reveals that initially cell growth is negligible for the first three hours, however between three and six hours the cells experience a period of rapid growth, which slowly tails off up to twelve hours. Around twelve hours the rate of cell growth begins to slowly decrease, maintaining this trend until the end of the experiment at 23 hours. Overlaid on the plot are the cell growth values obtained from the OD_600_ measurements, plotted as red squares. The OD_600_ values do not display such a smooth curve as there is an initial jump at one hour, then followed by negligible change until the five-hour mark at which point the values begin to increase. At 23 hours the OD_600_ values reveal a cell growth around x2, which is in agreement to the values from effective scattering parameter. The OD_600_ measurements are not as smooth as the line obtained from the optical scattering measurements. This difference is likely due to the fact that the optical measurements are averaged over the volume of the vessel and therefore are more representative of the system as a whole, while the OD_600_ measurements rely on manually taking 1 mL aliquots which are only representative of the local surroundings where the material is extracted from. The negative control measurement for E. coli with antibiotic is plotted in the blue line. The role of the antibiotic is to suppress the cell growth, and therefore we expect this measurement to show negligible cell growth. The blue line does show some small amount of growth, slowly increasing during the first ten hours, up to a maximum value ∼ 1.05. By the twelve hour mark this value then reverses its trend and begins to slowly decrease, reaching a final value ∼ 0.9 at 23 hours.

Both the E. coli cells, and negative control experiment, present a decrease in effective scattering parameter after the twelve-hour mark. This decrease is note-worthy; it is unsurprising that cell death will occur within the vessel, however the dead cells are still contained within the vessel, and could be expected to have a contribution to optical scattering. This apparent contradiction may be due to the difference in optical scattering parameters of live and dead cells; after death bacterial cells can change their structural properties which leads to a change in refractive index and scattering properties. Further, the dead cells can fall out of suspension and form agglomerates at the bottom of the vessel, which is outside of our optical measurement window.

Figure 5(b) plots the spatially resolved cell growth within the vessel for the E. coli cells. Two regions of erroneous data at 55 and 70 mm height are blanked out with white lines (see Supplement 1 S4) as it is observed that these locations produce spurious readings which are attributed to specular reflections from the vessel surface due to the coherent laser source. The spatially resolved colormap reveals that the cell growth is not uniform within the vessel. The top of the vessel at 30 mm displays a large band where there appears to be preferential cell growth, the middle region around 60 mm shows less growth, and then a region below at 90 mm again shows increased growth. This highlights the depth of information which can be obtained via spatially resolved optical measurements, which would not be available via spectrophotometer measurements on aliquots.

Figure 5(c) plots the spatially resolved values of cell growth for the *E. coli* cells with antibiotic. It is seen that during the 23 hour measurement period there is indeed negligible growth compared to the dataset without antibiotic, reaching a maximum growth of about 10 % at the twelve-hour mark. The results also reveal that, unlike the cells without antibiotic, there is negligible variation within the height of the vessel, suggesting the cellular distribution within the vessel remains fairly uniform for the duration of the experiment. As hypothesised, the negative control shows a negligible change in cell growth compared to the measurements without antibiotic, thereby confirming our optical measurement system is indeed working as expected.

### 3.3. Investigating influences of culture vessels on cell growth

The *in-situ* monitoring of cell growth in a bioreactor is technologically challenging due to physical constraints. Researchers typically rely on cell culture plates for small-scale experiments to monitor the growth dynamics of cell strains, utilising automated systems for characterising cell growth via optical density measurements. These small-scale experiments can provide indications of cell behaviour before production is scaled up, however it is known that cell behaviour is strongly influenced by the volume of growth vessels [25,26], and therefore any information gained from a well-plate must be viewed with caution when transferring this knowledge to different culturing environments. With the development of our optical measurement of cell density system we are able to record cell growth in an automated system over long measurement periods, which therefore provides equivalent data to OD_600_ measurements on a well plate but for a bioreactor. This method therefore allows us to investigate the influence of cell culture volumes on growth dynamics with real-time quantification of cell growth.

For this experiment, a cell line of *E. coli* was genetically-modified to include two fluorophore genes (GFP and mCherry). The fluorophores are not relevant to this current paper but are included for completeness; future versions of this system will incorporate fluorescent monitoring of cell health. It has been observed during our laboratory work that the growth dynamics of these particular strains of modified *E. coli* exhibited a response to glucose, which can be easily added without change to the optical properties of the system. For the bioreactor measurements we monitor the cell growth at a single fixed position within the vessel over a 23 hour period, for cells in media, and cells in media with added glucose. For each condition we repeat the experiment three times to obtain and mean and standard deviation for the cell growth factor. Additionally, a well plate measurement was performed on an identical strain of cells, both with and without glucose, to observe the growth dynamics at different culture volumes. For the well plate measurements we utilise two 96-well plates (one for the cells in media, and one for the cells in media with added glucose) with automated spectrophotometer measurements of OD_600_. Similarly, for the well plate measurements we calculate the mean and standard deviation for the cell growth factor.

**Fig. 6.**
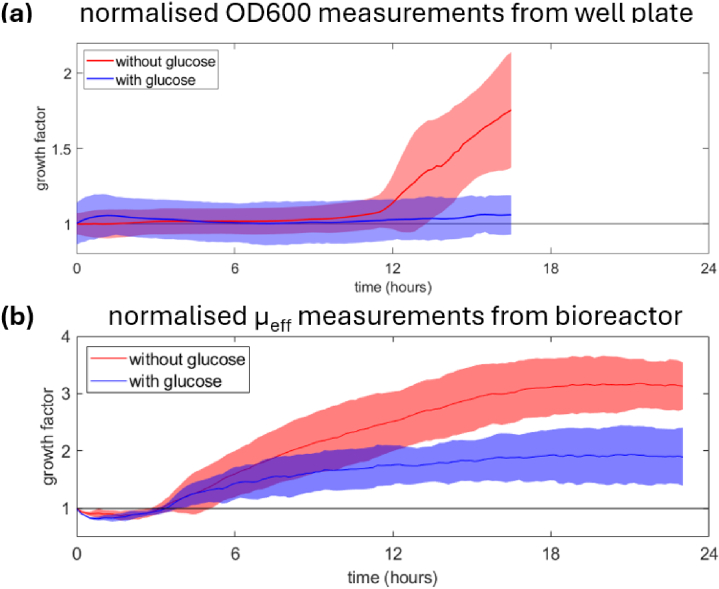
*E. coli* are cultured both with and without glucose to monitor effects on cell growth. **(a)** 96-well plate cell culture with cell growth monitored via OD_600_ spectrophotometer measurements. **(b)** Bioreactor cell culture with optical measurement of effective scattering parameter to monitor cell growth. In both scenarios it is seen that initially the growth with and without glucose shows an initially similar behaviour, however after several hours the batches without glucose start to grow more rapidly.

Figure 6(a) plots the cell growth for the well plate measurements, obtained for cells in media, and cells in media with added glucose, measured over a 17 hour period. The mean values are plotted with the solid red and blue lines, while the colour patch depicts the extent of the error bars, calculated as the standard deviation. For the cells without glucose, plotted in red, there is initially negligible variation in cell growth for the first 10 hours, at which point the growth starts to slowly rise, and then at the 12 hour mark the cell growth rate increases, and stays on an upward linear trend for the remaining duration of the measurement period. For the cells with added glucose, plotted in blue, there is an initial rise, then fall, in cell growth during the first 2 hours, however it is unclear whether this effect is real or a statistical artefact. The blue line then remains very flat, until the 12 hour mark at which point there is a slight upward trend. However, for the entire measurement period the results, the measured growth factor for the cells with added glucose do not show any statistically meaningful growth, as the extent of the error bars always encompass unity. Figure 6(b) presents the cell growth, obtained by optical measurements of effective scattering parameter, for the modified E. coli cells in the bioreactor vessel. The results reveal that for both cell growth conditions there is an initial decrease in cell growth, which is then recovered by 4 hours. After 4 hours the cells without glucose enter a growth phase which lasts until 18 hours, then slowly trails off at a maximum 3.2-fold increase in cell density at 23 hours. For the cells with added glucose the profile of cell growth is similar but smaller in magnitude, reaching a maximum 1.8-fold increase to cell density at 23 hours.

When comparing these sets of results the first observation is that in both culture vessels the addition of glucose into the system has the effect of either minimising, or completely preventing, the growth of the E. coli bacteria. For the well plate the addition of glucose appears to completely suppress the cell growth, while for the bioreactor vessel the growth is roughly halved compared to the cells without glucose. A clear difference is observed in the time at which cell growth begins depending on the culture vessel; for the well plate measurements there is negligible cell growth until 12 hours, however for the bioreactor vessel we observe cell growth after 4 hours. One likely contributing factor to these observed differences in cell growth in different culture vessels is the presence of mechanical stirring in the bioreactor by virtue of the impeller. Stirring of the bioreactor acts to homogenise the diffused gases and nutrients in the vessel, thereby removing respiration by-products and allowing for efficient cell growth. The well plates do not include any stirring mechanism and therefore may encounter a build-up of dissolved carbon dioxide.

Overall, our results demonstrate that, while there are clear parallels between the two culture vessels with the influence of glucose on cell growth, fundamental differences are observed between the well plates and the bioreactor even when using identical cell strains and growth media. These findings are an important consideration when scaling-up production from small to large scales. We envisage our optical measurement method can therefore be utilised to track changes in cell growth which are introduced by culture vessels during scale-up, thereby preventing researchers from erroneously relying on behaviours observed in well plates to influence their decisions when culturing larger volumes.

## 4. Conclusion

The culturing of cells in bioreactors is a very widely-used process in both the biological and chemical engineering sectors, however there are a lack of methods to monitor cell growth in a spatially-resolved manner, especially in-situ. Here we develop an optical method to infer local cell density by measurement of optical scattering parameters within the bioreactor vessel. Spatial information is obtained by guiding a laser beam around the surface of the vessel using galvo-scanning mirrors, and recording the light emitted from the vessel with a camera. We demonstrate how the effective scattering parameter scales linearly with cell density, and then utilise this method to monitor cell growth over extended time periods. Control measurements are presented using antibiotic agents to suppress growth, producing negligible change in scattering parameters. Unlike manual sampling of aliquots for analysis with a spectrophotometer, our method is capable of delivering spatially resolved information on cell growth and is fully automated for long-duration measurements. We believe that our measurement method is well-suited to investigating spatial heterogeneities in bioreactor vessels, and how these heterogeneities are affected by process control parameters such as agitation, temperature, and nutrient feeding. Further, we aim to incorporate these measurements into a feed-back loop with the bioreactor control unit, thereby creating an intelligent optimisation system, which will enhance the efficiency of bioreactors for bioprocessing applications. In future work we plan to investigate the potential use of genetically engineered fluorescent reporter genes, which are capable of tuning their fluorescence signal in response to cell-function or external stimuli such as lactate and oxygen concentrations.

## Supplemental information

Supplement 1 Supporting Information https://doi.org/10.6084/m9.figshare.31167364

## Data Availability

Data underlying the results presented in this paper are available at [27].

## Supplemental document

See Supplement 1 for supporting content.
