# Supplementary material for: Spatially-resolved optical monitoring of bioreactor cell growth
Source: Biomed Opt Express. 2026 Jan 30;17(2):1098–111. doi: 10.1364/BOE.583724 (PMC12904529; doi:10.1364/BOE.583724)
Supplement: Supplementary file 1 [file boe-17-2-1098-s001.pdf]

Supplemental Document

Biomedical Optics EXPRESS

Spatially-resolved optical monitoring of bioreactor cell growth: supplement

J. GORECKI,[1,*] C. REDWOOD-SAWYERR,[2] J. CAO,[3] H. DEHGHANI,[3] C. KONTORAVDI,[2] K. POLIZZI,[2] AND C. ROWLANDS[1]

[1] *Department of Bioengineering, Royal School of Mines, Imperial College London, SW7 2BX, UK*
[2] *Department of Chemical Engineering, Royal School of Mines, Imperial College London, SW7 2BX, UK*
[3] *School of Computer Science, University of Birmingham, B15 2TT, UK*
[*] *j.gorecki@imperial.ac.uk*

This supplement published with Optica Publishing Group on 30 January 2026 by The Authors under the terms of the Creative Commons Attribution 4.0 License in the format provided by the authors and unedited. Further distribution of this work must maintain attribution to the author(s) and the published article's title, journal citation, and DOI.

Supplement DOI: https://doi.org/10.6084/m9.figshare.31167364

Parent Article DOI: https://doi.org/10.1364/BOE.583724

Spatially-Resolved Optical Monitoring of Bioreactor Cell Growth
-Supporting Information-

J. Gorecki, C. Redwood-Sawyerr, J. Cao, H. Dehghani,
C. Kontoravdi, K. Polizzi C. Rowlands

Supplementary 1 - Image Unwrapping

The bioreactor is a cylindrical vessel which is imaged from a single fixed camera and lens position. The image formed on the camera is of the front surface of the bioreactor vessel, and reflections of the rear surfaces viewed from the two large mirrors. The aim of the image unwrapping algorithm is to take this camera image and reconstruct an image of the vessel surface as if it were able to be peeled off the surface and lain flat. The steps taken to construct this algorithm are detailed below.

The cylindrical vessel surface is wrapped in a sheet of paper upon which is printed a square grid with 1 cm spacing. Coordinates in the horizontal and vertical directions of the grid are labelled on the grid at 5 cm spacings to create a reference coordinate system, which are referred to as the 'Cartesian coordinates'. An image of the vessel covered in the paper grid is acquired from the camera, as shown in Figure S1(a). A script in Matlab is created in which the user manually identifies the locations of the Cartesian grid points (at 1 cm spacings) within the image. This process creates a pair-wise mapping between points on the paper grid (the Cartesian coordinates) and locations within the image (Image coordinates).

Figure S1(b) shows the manually selected grid points in the camera image. The vessel is split into three domains; left mirror, front surface, and right mirror, which are presented in green, red, and blue markers. For each of the three domains a Matlab function 'fitgeotform2D' is utilised which creates a coordinate transformation between the pair-wise list of Cartesian and Image coordinates. A 4th order polynomial is used for the transformation as this enables the transformation to produce curvature in the image, which is necessary for the cylindrical surface. Geometric transformations are created for the forward and reverse directions. The transformation is given the name Image2Cartesian as it takes locations within the image as an input, and outputs the coordinates in the Cartesian system. The transformation can also be inverted, which will convert Cartesian coordinates to locations within the image. By using a single transformation in the forward or inverse directions we ensure that any transformation is unique and single-valued, meaning that transforming from one system into the other, and then back to the original system, will reproduce the initial locations.

After the image transformations are created they are used to unwrap the image. A high-density mesh-grid (751 x 2053 pixels) is created in the Cartesian system. For each point, the Cartesian coordinates are converted into the Image coordinates and the pixel intensity evaluated at the point in the image. The unwrapped images are always viewed at the same 751 x 2053 resolution and therefore the transformation on this coordinate list only has to be undertaken once, as the list of pair-wise coordinates in Cartesian and Image systems is saved for future use, making the subsequent processing extremely fast. Figure S1(c) shows an example of an unwrapped image, taken when the vessel is wrapped in the paper grid, which is therefore the unwrapped version of Figure S1(a). The unwrapped image shows that the curvature of the grid has largely been removed, and the three disparate sections of the original image have been stitched together. There are artefacts visible where the sections stitch together which seem to result from variations in the image intensity near the edges of each domain.

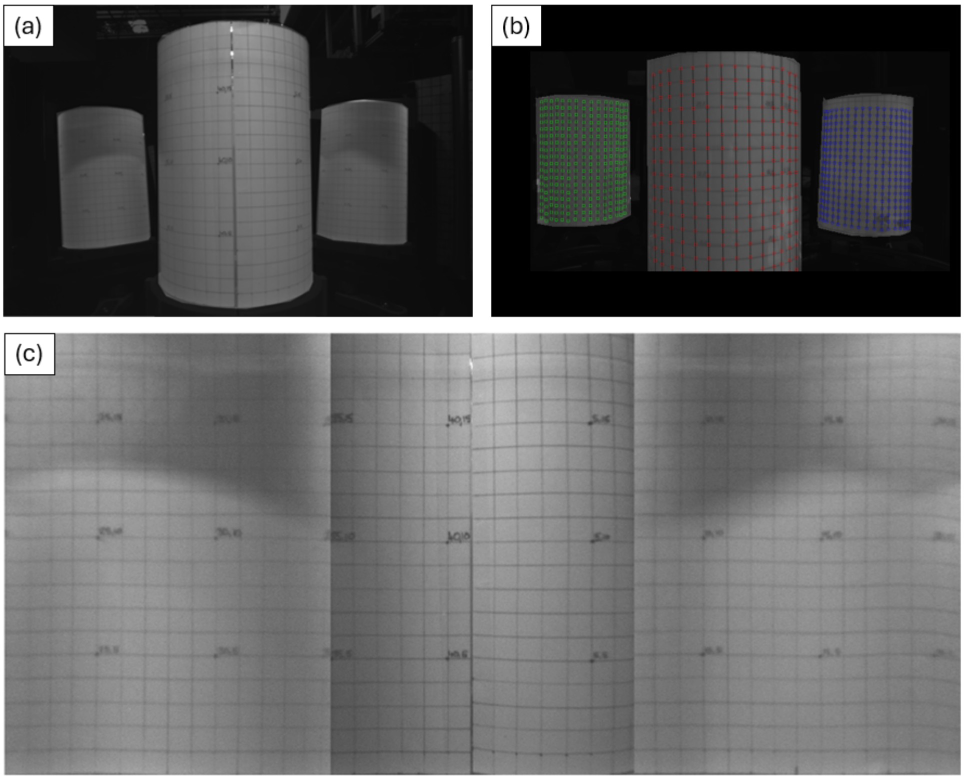

Figure S1: **(a)** The bioreactor vessel is wrapped in a sheet of paper printed with a 1 cm square grid. **(b)** The grid intersection points are manually selected in Matlab to locate their corresponding locations in the image coordinates, which are used to define the geometric transformation algorithm. **(c)** The image of the paper grid is unwrapped via the transformation algorithm to reproduce a flat grid obtained from a 360° view of the vessel.

Supplementary 2 - Laser Transformation

The direction of the laser beam is controlled by a pair of scanning galvo-mirrors. The angle of each mirror is controlled by a ± 10 volt signal. To illuminate the bioreactor vessel at any arbitrary point on its surface, we create a transformation algorithm which converts locations on the Cartesian grid (the flat grid on the surface of the vessel) to voltage values for the galvo-mirrors.

The galvo-mirrors are swept from - 10 to + 10 volts in 1 volt intervals. Each mirror therefore has 21 voltage positions, creating a list of 21^2 positions. For each position an image is acquired which reveals the location of the laser spot for the given voltage. Figure S2(a) shows the 441 images stacked together, revealing all the laser locations which were obtained. The three domains (left mirror, front surface, and right mirror) are isolated and analysed individually. All laser locations which are outside of these three domains are ignored. For each galvo voltage the location in image coordinates of the laser spot is identified, and then converted to Cartesian coordinates using the transformation Image2Cartesian. A geometric transformation is created to map the galvo-voltages to the Cartesian locations, where a separate transformation is utilised for each of the three domains. This transformation is called Volt2Cartesian, and can be used both in the forward and inverse directions.

To demonstrate the Volt2Cartesian transformation we generate a list of Cartesian coordinates (at the intersection points on the 1 cm grid) and transform these coordinates to voltages. The voltage values are plotted in Figure S2(b). To confirm the validity of this transformation, we pass these voltage values into the galvo-mirror pair and acquire an image at each voltage value. The images are stacked together as shown in Figure S2(c) which reveals that the laser has indeed been directed to each intersection point on the 1 cm Cartesian grid on the surface of the vessel.

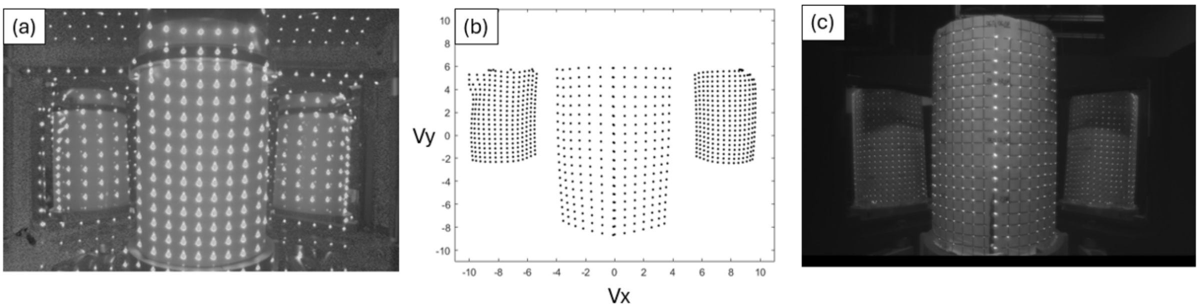

Figure S2: **(a)** The galvo-mirrors are each swept from -10 to +10 volts in 1 volt increments, acquiring an image at each voltage value. **(b)** Galvo-mirror voltage values are obtained from the inverse of the Volt2Cartesian transformation **(c)** The voltage values are input to the galvo-mirror paid and an image acquired at each value, demonstrating the successful steering of the laser towards the intersection points on the 1 cm Cartesian grid.

Supplementary 3 - HDR Imaging

The image intensity varies over a broad set of values, and therefore we capture high dynamic range (HDR) images. The HDR algorithm combines a set of low dynamic-range pixels to make a high dynamic-range pixel. It is assumed that within the set of low dynamic-range pixels there will be several exposure values which produced a well exposed pixel, several which produced an under-exposed pixel, and several which produced an over-exposed pixel. The goal of the HDR algorithm is essentially to find the exposure values which produced the well-exposed pixels and perform an averaging. A weighting function is applied such that the under-exposed and over-exposed pixels have negligible contribution to the averaged value, as they hold little useful information. The camera pixel values are 12-bit, and therefore range from 0 to $2^{12} - 1$, (4095). A Tukey window is used for the weighting, which is a tapered cosine window, with a value of 0.4 as the smoothness coefficient. Figure S3(a) shows the weight as a function of pixel value, which determines how much it contributes to the averaging.

For a series of low dynamic-range pixels in a given location, the value of the high dynamic-range pixel **L** is given by equation 1, where **N** is the number of low dynamic-range exposures, **Z** is the pixel value of the low dynamic-range image, $\mathbf{w(Z{+}1)}$ is the weight of the pixel (adjusting for the index starting from one instead of zero), and **t** is the exposure time.

$$L = \sum_{k=1}^{N} \frac{Z\ w(Z+1)}{t} / \sum_{k=1}^{N} w(Z+1) \tag{1}$$

The camera is capable of achieving exposure times between 28 μs and 2 s. We chose a series of exposure times which covers almost the full range of permissible values, starting from a minimum value of 28 μs, using a geometric series where each successive value is the previous value multiplied by 16, up to a maximum of 1.8 seconds. A total of 5 exposure times are utilised. This selection of exposure times ensures that there is sufficient overlap between the successive exposures, while maintaining a low total number of exposures.

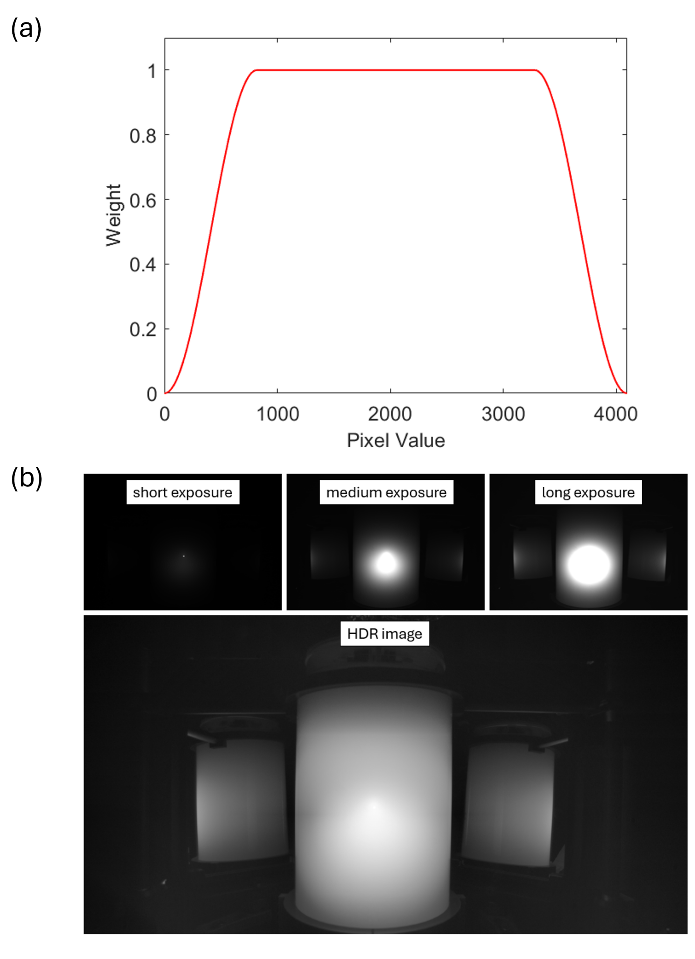

Figure S3: **(a)** Pixel Weighting Function. **(b)** An example of three low-dynamic-range images of the bioreactor vessel illuminated by a single laser spot. Each low-dynamic-range image contains significant portions where pixels are either under- or over- exposed. The three images are combined to form a high-dynamic-range image which extends to bit-depth of the image to avoid under- or over-exposure of pixels.

Supplementary 4 - Erroneous Scattering Data

The spatially resolved measurements of effective scattering parameter produced erroneous data at two consistent heights in all measurements, where values are outside of the expected range. It is hypothesised that these values are influenced by specular reflections from the surface of the vessel being redirected back into the imaging system, and do not reproduce scattering information from the interior of the vessel. To confirm this hypothesis we perform spatially resolved measurements of the effective scattering parameters within the vessel, while manually raising the bioreactor vessel to a range of predefined heights. The vessel is filled with a dilution of milk and water to create a weakly scattering sample with homogeneous mixing. Figure S4 plot the spatially resolved scattering parameters within the vessel for a range of vertical offsets (for example, a value of Δy corresponds to be bioreactor vessel being manually raised by 53 mm as compared to its normal position.) The results reveal that for the initial displacement of $\Delta y = 0$ there is a mostly flat profile to the scattering parameter, except for two dips which appear close to 55 and 70 mm in height. When the vessel is vertically displaced we observe that these dips do not change position, which would be the case if they were due to the internal contents of the vessel. As the peaks remain in fixed vertical positions, despite moving the vessel, we must conclude these are artefacts and are likely due to specular reflection from the vessel surface.

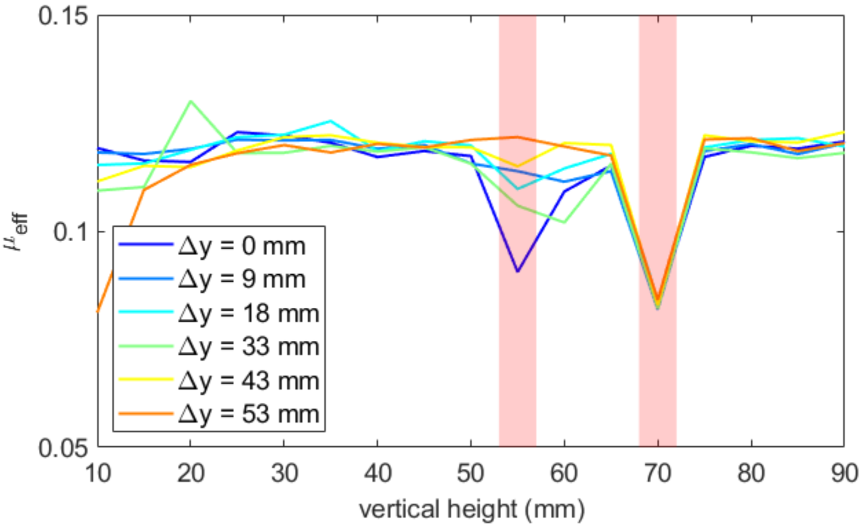

Figure S4: Spatially resolved scattering parameters measured while manually adjusting the vertical position of the bioreactor vessel. The two peaks in the data observed at 50 and 65 mm remain stationary compared to the vessel displacement, therefore suggesting data at these locations are erroneous and not linked to the interior conditions of the vessel.

Supplementary 5 - OD_{600} Measurement Calibration

Experimental data provided in Figure 5a observed OD_{600} measurements with a range from a minimum of 0.88 to 1.77 at maximum. To confirm that the OD_{600} measurements are linearly proportional to cell density, within the measurement range used in experiments, a serial dilution calibration is performed. A stock solution of *E. coli* cells is diluted to a range of cell density values. Optical density measurements are performed on a Jenway 6305 spectrophotometer, utilising Brand^{TM} Polystyrene Semi-Micro cuvettes. The spectrophotometer is set to a measurement wavelength of 600 nm and blanked against a cuvette of cell media. For each sample of the serial dilution, 1 mL of solution is transferred with a micro-pipette into a new cuvette. Figure S5 shows the measured value of OD_{600} plotted against the cell density, which is provided in arbitrary units. A linear fit is provided to the data with the intercept fixed at the origin, and the fit quantified by R-squared value.

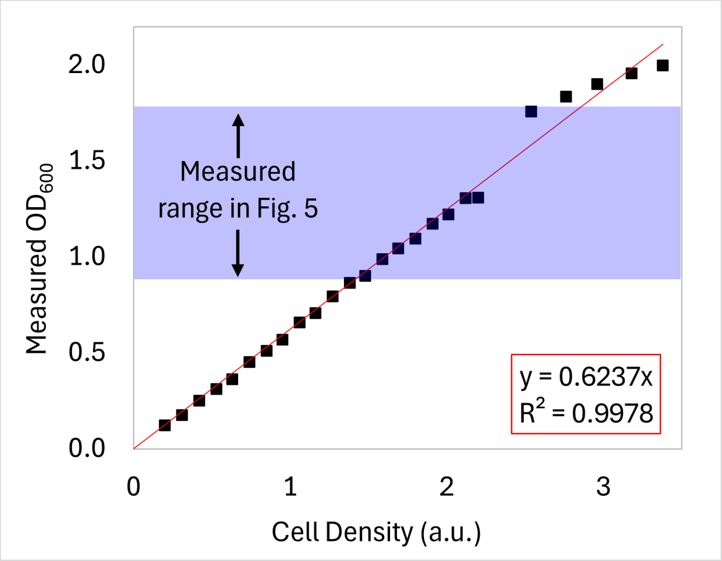

Figure S5: OD calibration graph

Supplementary 6 - Camera Sensor Noise

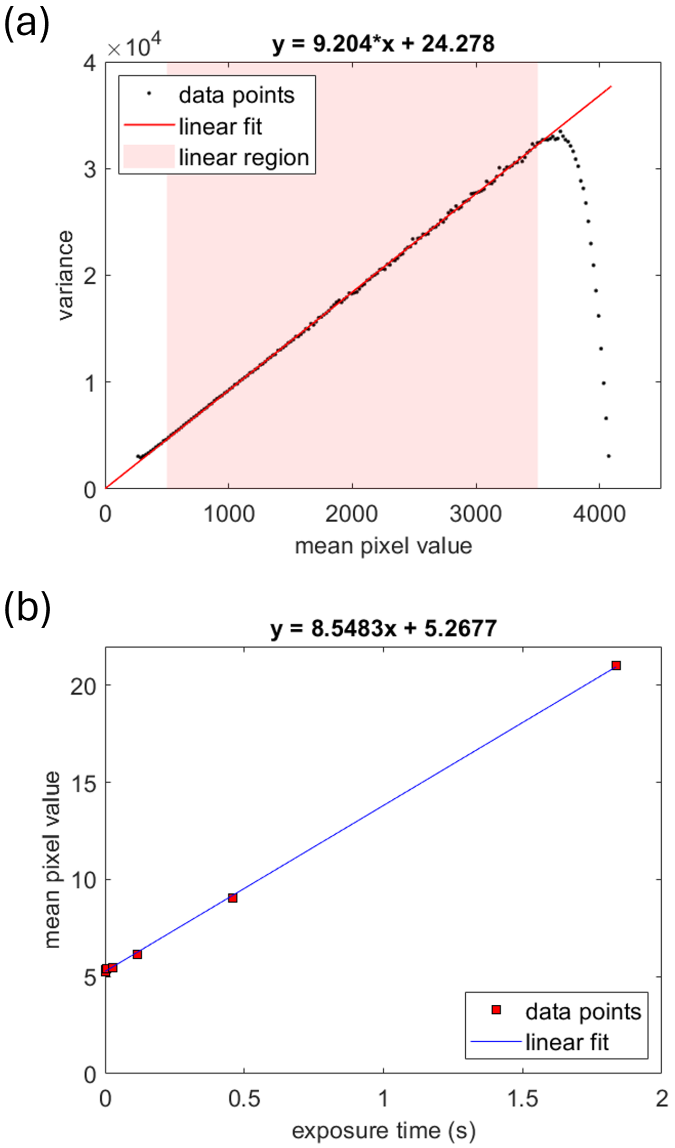

Figure S6: Sensor noise characterisation.

Sensor noise is characterised by analysing the variance in a set of static images. A series of 40 images are acquired from a static scene. The temporal variance method is utilised whereby the mean value and variance for each pixel are calculated across the image stack. These values are then binned into 200 evenly spaced bins covering pixel intensities from 0 to 4095, and averaged. The resulting data is displayed as a plot of variance against mean pixel value in Figure S6(a), which shows a linear relationship which breaks down at high pixel values.

The linear region corresponds to the shot-noise-limited regime, where shot noise dominates over read noise and the sensor response is linear. In this region variance is proportional to the mean signal, consistent with Poisson statistics, however, due to the internal

gain in the sensor, the gradient is not unity. From this dataset we obtain a gradient of 9.2 which corresponds to the gain in the system and therefore reveals that the pixel values on the sensor must be multiplied by this number to convert to electron numbers. Finally, as we are operating within the Poisson-noise limited region, the standard deviation in the signal can be determined from the square root of the electron number, and the standard deviation of the read noise is obtained from the square root of the y-axis intercept from the linear fit.

Camera Black Level is characterised in Figure S6(b) below by recording images at a range of exposure values without any laser illumination within the light proof box. A linear fit is applied to the data. The camera black level is obtained from the y-axis intercept which provides the average camera reading at an exposure time of zero seconds. From this analysis we find the camera black level to have a value of 5.3, given in units of Digital Number.
